# Supplementary material for: First-Trimester Influenza Infection Increases the Odds of Non-Chromosomal Birth Defects: A Systematic Review and Meta-Analysis
Source: Viruses. 2022 Dec 2;14(12):2708. doi: 10.3390/v14122708 (PMC9781815; doi:10.3390/v14122708)
Supplement: Supplementary file 1 [file viruses-14-02708-s001.zip › viruses-1992690-supplementary.pdf]

First-Trimester Influenza Infection Increases the Odds of Non-Chromosomal Birth Defects: A Systematic Review and Meta-Analysis

Ákos Mátrai [1,2,3], Brigitta Teutsch [2,4], Alex Váradi [4], Péter Hegyi [2,4,5], Boglárka Pethő [1,2], Akari Fujisawa [2,6], Szilárd Váncsa [2,4,5], Balázs Lintner [1,2], Zsolt Melczer [1,2] and Nándor Ács [1,2,*]

1 Department of Obstetrics and Gynecology, Semmelweis University, 1085 Budapest, Hungary
2 Centre for Translational Medicine, Semmelweis University, 1085 Budapest, Hungary
3 Faculty of Health Sciences, University of Pécs, 7621 Pécs, Hungary
4 Institute for Translational Medicine, Medical School, University of Pécs, 7621 Pécs, Hungary
5 Division of Pancreatic Diseases, Heart and Vascular Center, Semmelweis University, 1085 Budapest, Hungary
6 Faculty of Medicine, Semmelweis University, 1085 Budapest, Hungary
* Correspondence: acsnandor@gmail.com; Tel.: +36-309520861

Tables

Table S1. PRISMA 2020 checklist

Table S2. MOOSE Checklist for Meta-analyses of Observational Studies

Table S3. Table of included studies with outcomes

Table S4. The odds ratios for developing specific types of congenital malformations after first trimester

Table S5. Quality of evidence using GRADEPro

Figure legends

Figure S1. Forest plot representing that the odds for developing specific types of neural tube defects was increased after influenza in the first trimester.

Figure S2. Forest plot representing that the adjusted odds for developing limb reduction defects was increased after influenza in the first trimester.

Figure S3. Forest plot representing that the adjusted odds for developing eye anomalies was increased after influenza in the first trimester.

Figure S4. Risk of bias assessment at study and at domain level for all neural tube defects

Figure S5. Risk of bias assessment at study and at domain level for neural tube defects (mix)

Figure S6. Risk of bias assessment at study and at domain level for spina bifida

Figure S7. Risk of bias assessment at study and at domain level for cleft lip and palate

Figure S8. Risk of bias assessment at study and at domain level for cleft lip, palate

Figure S9. Risk of bias assessment at study and at domain level for congenital heart disease (adjusted, mix)

Figure S10. Risk of bias assessment at study and at domain level for congenital heart disease (adjusted)

Figure S11. Risk of bias assessment at study and at domain level for congenital heart disease (unadjusted, mix)

Figure S12. Risk of bias assessment at study and at domain level for congenital heart disease (unadjusted)

Figure S13. Risk of bias assessment at study and at domain level for eye anomalies

Figure S14. Risk of bias assessment at study and at domain level for limb reduction

Figure S15. Search key of the systematic search

Table S1. PRISMA 2020 checklist

Section and Topic	Item #	Checklist item	Location where item is reported
**TITLE**			
Title	1	Identify the report as a systematic review.	1-4
**ABSTRACT**			
Abstract	2	See the PRISMA 2020 for Abstracts checklist.	20-33
**INTRODUCTION**			
Rationale	3	Describe the rationale for the review in the context of existing knowledge.	37-68
Objectives	4	Provide an explicit statement of the objective(s) or question(s) the review addresses.	69-76
**METHODS**			
Eligibility criteria	5	Specify the inclusion and exclusion criteria for the review and how studies were grouped for the syntheses.	94-110
Information sources	6	Specify all databases, registers, websites, organisations, reference lists and other sources searched or consulted to identify studies. Specify the date when each source was last searched or consulted.	87-89
Search strategy	7	Present the full search strategies for all databases, registers and websites, including any filters and limits used.	89-92, Supplementary Material
Selection process	8	Specify the methods used to decide whether a study met the inclusion criteria of the review, including how many reviewers screened each record and each report retrieved, whether they worked independently, and if applicable, details of automation tools used in the process.	94-103, Supplementary Material
Data collection process	9	Specify the methods used to collect data from reports, including how many reviewers collected data from each report, whether they worked independently, any processes for obtaining or confirming data from study investigators, and if applicable, details of automation tools used in the process.	111-119
Data items	10a	List and define all outcomes for which data were sought. Specify whether all results that were compatible with each outcome domain in each study were sought (e.g. for all measures, time points, analyses), and if not, the methods used to decide which results to collect.	99-119
	10b	List and define all other variables for which data were sought (e.g. participant and intervention characteristics, funding sources). Describe any assumptions made about any missing or unclear information.	99-119
Study risk of bias assessment	11	Specify the methods used to assess risk of bias in the included studies, including details of the tool(s) used, how many reviewers assessed each study and whether they worked independently, and if applicable, details of automation tools used in the process.	143-148
Effect measures	12	Specify for each outcome the effect measure(s) (e.g. risk ratio, mean difference) used in the synthesis or presentation of results.	121-141
Synthesis methods	13a	Describe the processes used to decide which studies were eligible for each synthesis (e.g. tabulating the study intervention characteristics and comparing against the planned groups for each synthesis (item #5)).	121-141

Section and Topic	Item #	Checklist item	Location where item is reported
	13b	Describe any methods required to prepare the data for presentation or synthesis, such as handling of missing summary statistics, or data conversions.	121-141
	13c	Describe any methods used to tabulate or visually display results of individual studies and syntheses.	121-141
	13d	Describe any methods used to synthesize results and provide a rationale for the choice(s). If meta-analysis was performed, describe the model(s), method(s) to identify the presence and extent of statistical heterogeneity, and software package(s) used.	121-141
	13e	Describe any methods used to explore possible causes of heterogeneity among study results (e.g. subgroup analysis, meta-regression).	121-141
	13f	Describe any sensitivity analyses conducted to assess robustness of the synthesized results.	121-141
Reporting bias assessment	14	Describe any methods used to assess risk of bias due to missing results in a synthesis (arising from reporting biases).	121-141
Certainty assessment	15	Describe any methods used to assess certainty (or confidence) in the body of evidence for an outcome.	149-152
**RESULTS**			
Study selection	16a	Describe the results of the search and selection process, from the number of records identified in the search to the number of studies included in the review, ideally using a flow diagram.	154-173
	16b	Cite studies that might appear to meet the inclusion criteria, but which were excluded, and explain why they were excluded.	154-173
Study characteristics	17	Cite each included study and present its characteristics.	Table 1
Risk of bias in studies	18	Present assessments of risk of bias for each included study.	262-268, Supplementary Material Figure S4-S14
Results of individual studies	19	For all outcomes, present, for each study: (a) summary statistics for each group (where appropriate) and (b) an effect estimate and its precision (e.g. confidence/credible interval), ideally using structured tables or plots.	176-261
Results of syntheses	20a	For each synthesis, briefly summarise the characteristics and risk of bias among contributing studies.	176-261
	20b	Present results of all statistical syntheses conducted. If meta-analysis was done, present for each the summary estimate and its precision (e.g. confidence/credible interval) and measures of statistical heterogeneity. If comparing groups, describe the direction of the effect.	176-261
	20c	Present results of all investigations of possible causes of heterogeneity among study results.	176-261
	20d	Present results of all sensitivity analyses conducted to assess the robustness of the synthesized results.	176-261
Reporting biases	21	Present assessments of risk of bias due to missing results (arising from reporting biases) for each synthesis assessed.	262-268, Supplementary Material Figure S4-S14

Section and Topic	Item #	Checklist item	Location where item is reported
Certainty of evidence	22	Present assessments of certainty (or confidence) in the body of evidence for each outcome assessed.	267-268, Supplementary Material Table S5
**DISCUSSION**			
Discussion	23a	Provide a general interpretation of the results in the context of other evidence.	269-330
	23b	Discuss any limitations of the evidence included in the review.	331-343
	23c	Discuss any limitations of the review processes used.	331-343
	23d	Discuss implications of the results for practice, policy, and future research.	344-351
**OTHER INFORMATION**			
Registration and protocol	24a	Provide registration information for the review, including register name and registration number, or state that the review was not registered.	82-85
	24b	Indicate where the review protocol can be accessed, or state that a protocol was not prepared.	82-85
	24c	Describe and explain any amendments to information provided at registration or in the protocol.	85
Support	25	Describe sources of financial or non-financial support for the review, and the role of the funders or sponsors in the review.	369
Competing interests	26	Declare any competing interests of review authors.	376
Availability of data, code and other materials	27	Report which of the following are publicly available and where they can be found: template data collection forms; data extracted from included studies; data used for all analyses; analytic code; any other materials used in the review.	357-358, 374-375

From: Page MJ, McKenzie JE, Bossuyt PM, Boutron I, Hoffmann TC, Mulrow CD, et al. The PRISMA 2020 statement: an updated guideline for reporting systematic reviews. BMJ 2021;372:n71. doi: 10.1136/bmj.n71

For more information, visit: http://www.prisma-statement.org/

Table S2. MOOSE Checklist for Meta-analyses of Observational Studies

Item No	Recommendation	Reported on Page No
	Reporting of background should include	
1	Problem definition	1-2
2	Hypothesis statement	2
3	Description of study outcome(s)	2-3
4	Type of exposure or intervention used	2-3
5	Type of study designs used	2-3
6	Study population	2-3
	Reporting of search strategy should include	
7	Qualifications of searchers (eg, librarians and investigators)	3
8	Search strategy, including time period included in the synthesis and key words	3, Supplementary Material Appendix S1
9	Effort to include all available studies, including contact with authors	3
10	Databases and registries searched	2
11	Search software used, name and version, including special features used (eg, explosion)	2
12	Use of hand searching (eg, reference lists of obtained articles)	3
13	List of citations located and those excluded, including justification	3
14	Method of addressing articles published in languages other than English	3
15	Method of handling abstracts and unpublished studies	3
16	Description of any contact with authors	3
	Reporting of methods should include	
17	Description of relevance or appropriateness of studies assembled for assessing the hypothesis to be tested	3
18	Rationale for the selection and coding of data (eg, sound clinical principles or convenience)	3
19	Documentation of how data were classified and coded (eg, multiple raters, blinding and interrater reliability)	3
20	Assessment of confounding (eg, comparability of cases and controls in studies where appropriate)	3
21	Assessment of study quality, including blinding of quality assessors, stratification or regression on possible predictors of study results	3-4
22	Assessment of heterogeneity	3
23	Description of statistical methods (eg, complete description of fixed or random effects models, justification of whether the chosen models account for predictors of study results, dose-response models, or cumulative meta-analysis) in sufficient detail to be replicated	3
24	Provision of appropriate tables and graphics	3
	Reporting of results should include	
25	Graphic summarizing individual study estimates and overall estimate	9-12
26	Table giving descriptive information for each study included	6
27		
28	Indication of statistical uncertainty of findings	12, Supplementary

Material Figure S4-S14

Table S3. Table of included studies with outcomes

Author, year	Congenital malformations
L. Ács et al. 2020[1]	Cleft palate
Ács et al. 2005[2]	Neural tube defects; Cleft lip/palate; Cleft palate; Esophageal atresia; Pyloric stenosis; Intestinal atresia/ stenosis; Rectal/anal stresia/stenosis; Renal a/dysgenesia; Obstructive urinary Cas; Hypospadiasis; Undescended testis; Exomphalos/gastroschisis; Congenital hydrocephaly, Ear CAs, Cardiovascular CAs, Clubfoot, Limb reduction defects, Poly/syndactilia, Diaphragmatic CAs, Other Isolated CAs, Multiple CAs
Aro et al. 1983[3]	limb reduction defects
Botto et al. 2001[4]	Congenital heart defects, Transposition of great arteries, Tetralogy of Fallot, Atrioventricular septal defect, Ebstein anomaly, Anomalous pulmonary venous return, All right obstructive defects, Tricuspid atresia, All left obstructive defects, Hypoplastic left heart, Aortic stenosis, Aortic coarctation, Ventricular septal defect, Atrial septal defect
Busby et al. 2005[5]	Anophtalmia, Micophtalmia
Czeizel et al. 2008 [6]	Neural-tube defects, Anencephalus+-spina bifida, Spina bifida aperta/cystica, Encephalocele, occipital, Microcephaly, primary, Congenital hydrocephalus, CAs of eye, Anophthalmia–microphthalmia, Primary congenital glaucoma, Congenital cataract, Ocular coloboma, CAs of ear, Auditory canal+ear Cas, An/microtia, Others, unspecified, Cardiovascular CAs, Transposition of great vessels, Ventricular septal defect, Atrial septal defect, type II, Hypoplastic left heart, Patent ductus arteriosus, CAs of aorta, CAs of pulmonary valve, Others or unspecified, Brachial cyst, cleft, fistula, preauricular sinus, CAs of respiratory system, Cleft palate, Robin sequence, Cleft lip+-cleft palate, Cleft lip, Cleft lip with palate, Esophageal atresia/stenosis with or without tracheoesophageal fistula, Cong hypertrophic pyloric stenosis, Atresia/stenosis of small intestine, Atresia/stenosis of rectum/anal canal, Other CAs of digestive system, Hirschprung's disease, Other CAs of intestine, Other CAs of digestive system, Undescended testis (diagnosed after 3rd postnatal month), Hypospadias (without coronal type), Other CAs of genital organs, Renal a/dysgenesis,Obstructive CAs of urinary tract, Cystic kidney (diseases), Obstructive CAs of renal pelvis and ureter (hydronephrosis, constricture of ureteropelvic junction and ureterovesical orifice) , Other CAs of urinary tract, Other CAs of kidney, Other CAs of bladder and urethrea, Clubfoot, Poly/syndactyly,Polydactyly,Syndactyly (without minor), Limb deficiencies, Other CAs of limbs,

	CAs of diaphragm, CAs of abdominal wall (exomphalos and gastroschisis are not differentiated), Multiple CAs (major gene mutations and chromosomal aberrations are excluded)
Dymanus et al. 2019[7]	Cleft lip
Granroth 1978[8]	Anencephalia, Spina bifida, Congenital hydrocephaly, Microcephaly, Hydrancephaly, Polydactylia
Li et al. 2014[9]	All congenital heart defects, Septal defects, Conotruncal defects, Right-sided obstructive defects, Left-sided obstructive defects, Anomalous pulmonary venous return, Other isolated CAs
Lynberg et al. 1994[10]	Anencephalia, Spina bifida, Encephalocele
Ou et al. 2015[11]	Cardiovascular CAs, Ventricular septal defect, Atrial septal defect, Pulmonary stenosis, Dextro-transposition of great arteries, Tetralogy of Fallot
Park et al. 1993[12]	Anencephalia, Spina bifida
Saxen et al. 1975[13]	Cleft lip and palate
Saxen et al. 1975[14]	Cleft lip and palate

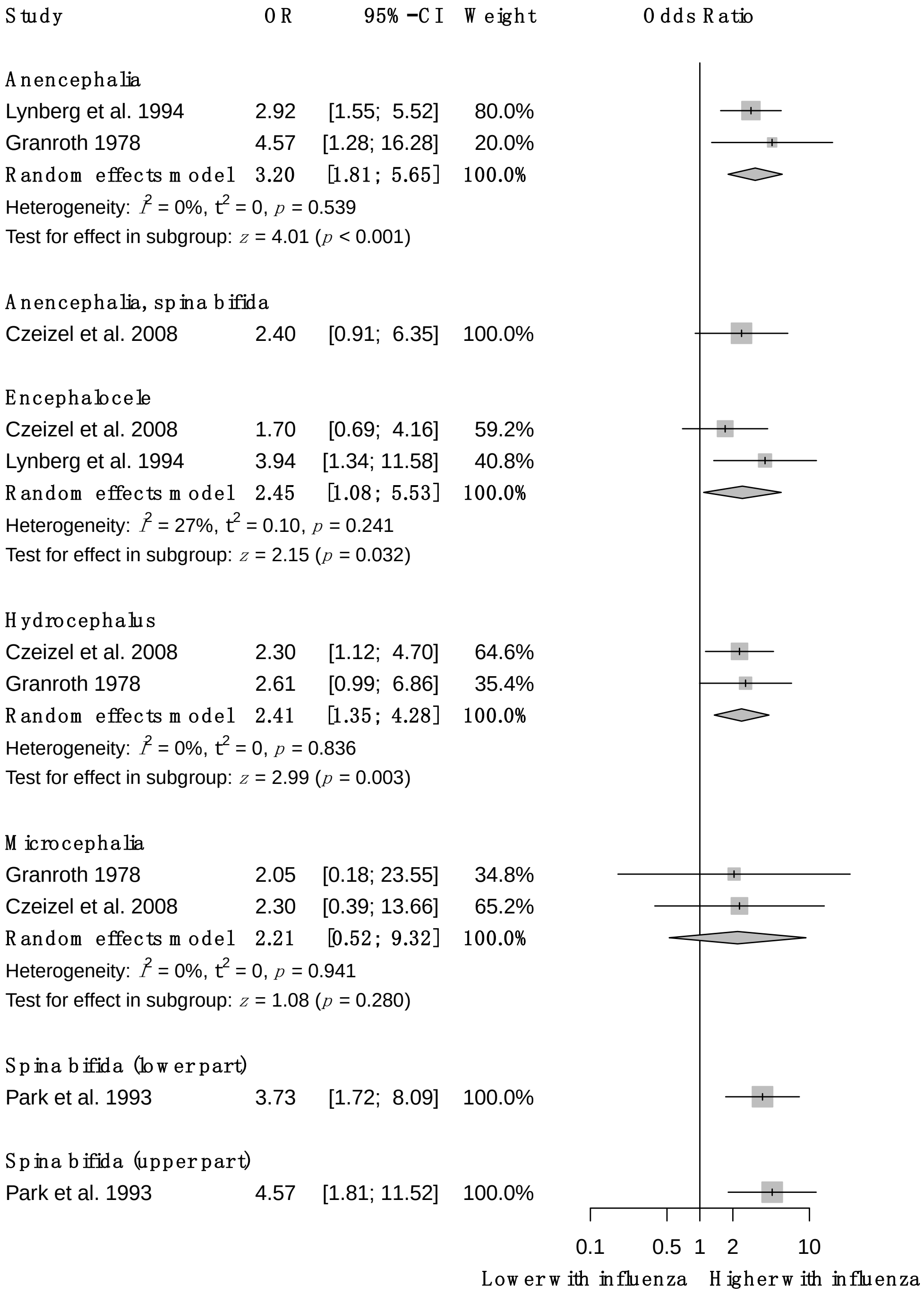

Figure S1. Forest plot representing that the odds for developing specific types of neural tube defects was increased after influenza in the first trimester.

Table S4. The odds ratios for developing specific types of congenital malformations after first trimester

Author, year	Congenital malformations	Odd ratios
Ács et al 2005 [2]	Esophageal atresia	1.20 (0.20−6.30)
Ács et al 2005 [2]	Pyloric stenosis	2.10 (0.20−7.00)
Ács et al 2005 [2]	Intestinal atresia/ stenosis	3.00 (0.60−13.60)
Ács et al 2005 [2]	Rectal/anal stresia/stenosis	0.70 (0.20−3.00)
Ács et al 2005 [2]	Renal a/dysgenesia	1.50 (0.20−10.00)
Ács et al 2005 [2]	Obstructive urinary Cas	1.20 (0.40−3.60)
Ács et al 2005 [2]	Hypospadiasis	1.00 (0.70−1.40)
Ács et al 2005 [2]	Undescended testis	0.70 (0.40−1.20)
Ács et al 2005 [2]	Exomohalos/gastrischisis	1.70 (0.50−5.10)
Ács et al 2005 [2]	Clubfoot	1.10 (0.70−1.70)
Ács et al 2005 [2]	Poly/syndactilia	1.00 (0.60−1.60)
Ács et al 2005 [2]	Diaphragmatic Cas	1.40 (0.40−4.50)
Ács et al 2005 [2]	Other isolated CAs	1.30 (0.80−2.20)
Ács et al 2005 [2]	Multiple CAs	1.50 (0.80−2.60)
Ács et al 2005 [2]	Total cases	1.40 (1.30−1.60)
Czeizel et al. 2008 [6]	Primary congenital glaucoma	2.00 (0.20−22.00)
Czeizel et al. 2008 [6]	Congenital cataract	3.00 (0.60−14.90)
Czeizel et al. 2008 [6]	Ocular coloboma	
Czeizel et al. 2008 [6]	Brachial cyst, cleft, fistula, preauricular sinus	0.90 (0.10−10.30)
Czeizel et al. 2008 [6]	CAs of respiratory system	
Czeizel et al. 2008 [6]	Cleft palate	2.50 (1.40−4.30)
Czeizel et al. 2008 [6]	Robin sequence	2.40 (0.20−39.7)

Czeizel et al. 2008 [6]	Cleft lip+-cleft palate	2.90 (2.10−4.10)
Czeizel et al. 2008 [6]	Cleft lip	2.40 (1.40−4.00)
Czeizel et al. 2008 [6]	Cleft lip with palate	3.40 (2.20−5.30)
Czeizel et al. 2008 [6]	Esophageal atresia/stenosis with or without tracheoesophageal fistula	1.70 (0.60−4.80)
Czeizel et al. 2008 [6]	Cong hypertrophic pyloric stenosis	1.10 (0.40−3.00)
Czeizel et al. 2008 [6]	Atresia/stenosis of small intestine	5.30 (1.40−20.20)
Czeizel et al. 2008 [6]	Atresia/stenosis of rectum/anal canal	0.90 (0.30−2.40)
Czeizel et al. 2008 [6]	Other CAs of digestive system	2.70 (1.10−6.60)
Czeizel et al. 2008 [6]	Hirschprung's disease	3.00 (0.30−35.80)
Czeizel et al. 2008 [6]	Other Cas of intestine	2.50 (0.90−7.30)
Czeizel et al. 2008 [6]	Other CAs of digestive system	3.20 (0.30−36.60)
Czeizel et al. 2008 [6]	Undescended testis (diagnosed after 3rd postnatal month)	0.50 (0.30−0.80)
Czeizel et al. 2008 [6]	Hypospadias (without coronal type)	1.00 (0.80−1.40)
Czeizel et al. 2008 [6]	Other CAs of genital organs	2.30 (0.40−12.50)
Czeizel et al. 2008 [6]	Renal a/dysgenesis	1.00 (0.30−3.40)
Czeizel et al. 2008 [6]	Obstructive CAs of urinary tract	1.30 (0.60−3.10)
Czeizel et al. 2008 [6]	Cystic kidney (diseases)	1.60 (0.60−4.30)
Czeizel et al. 2008 [6]	Obstructive CAs of renal pelvis and ureter	0.80 (0.10−4.30)

	(hydronephrosis, constricture of ureteropelvic junction and ureterovesical orifice)	
Czeizel et al. 2008 [6]	Other CAs of urinary tract	2.40 (0.90−6.40)
Czeizel et al. 2008 [6]	Other Cas of kidney	2.10 (0.70−6.30)
Czeizel et al. 2008 [6]	Other Cas of bladder and urethrea	4.00 (0.40−44.10)
Czeizel et al. 2008 [6]	Clubfoot	1.30 (0.90−1.80)
Czeizel et al. 2008 [6]	Poly/syndactyly	1.10 (0.70−1.70)
Czeizel et al. 2008 [6]	Polydactyly	1.00 (0.60−1.70)
Czeizel et al. 2008 [6]	Syndactyly (without minor)	1.30 (0.70−2.60)
Czeizel et al. 2008 [6]	Limb deficiencies	2.30 (1.30−4.10)
Czeizel et al. 2008 [6]	Other CAs of limbs	0.30 (0.00−2.70)
Czeizel et al. 2008 [6]	CAs of diaphragm	3.80 (1.40−10.20)
Czeizel et al. 2008 [6]	CAs of abdominal wall (exomphalos and gastroschisis are not differentiated)	1.50 (0.60−3.80)
Czeizel et al. 2008 [6]	Multiple CAs (major gene mutations and chromosomal aberrations are excluded)	2.20 (1.50−3.20)

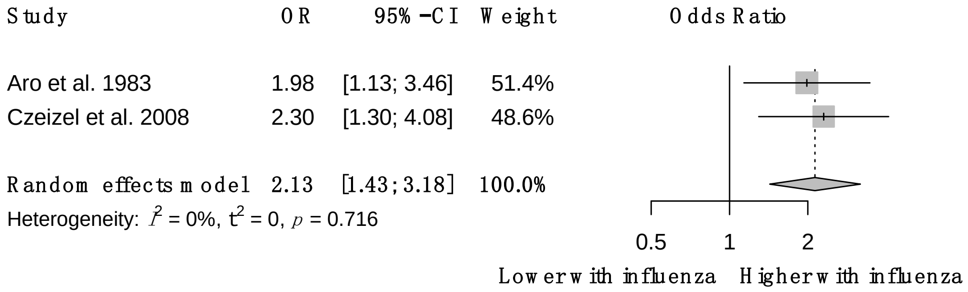

Figure S2. Forest plot representing that the adjusted odds for developing limb reduction defects was increased after influenza in the first trimester.

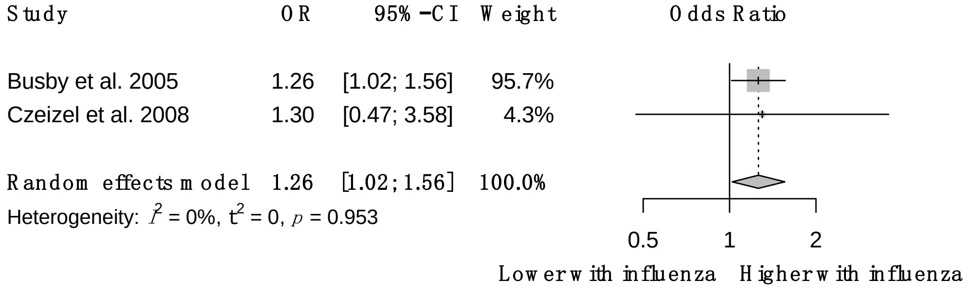

Figure S3. Forest plot representing that the adjusted odds for developing eye anomalies was increased after influenza in the first trimester.

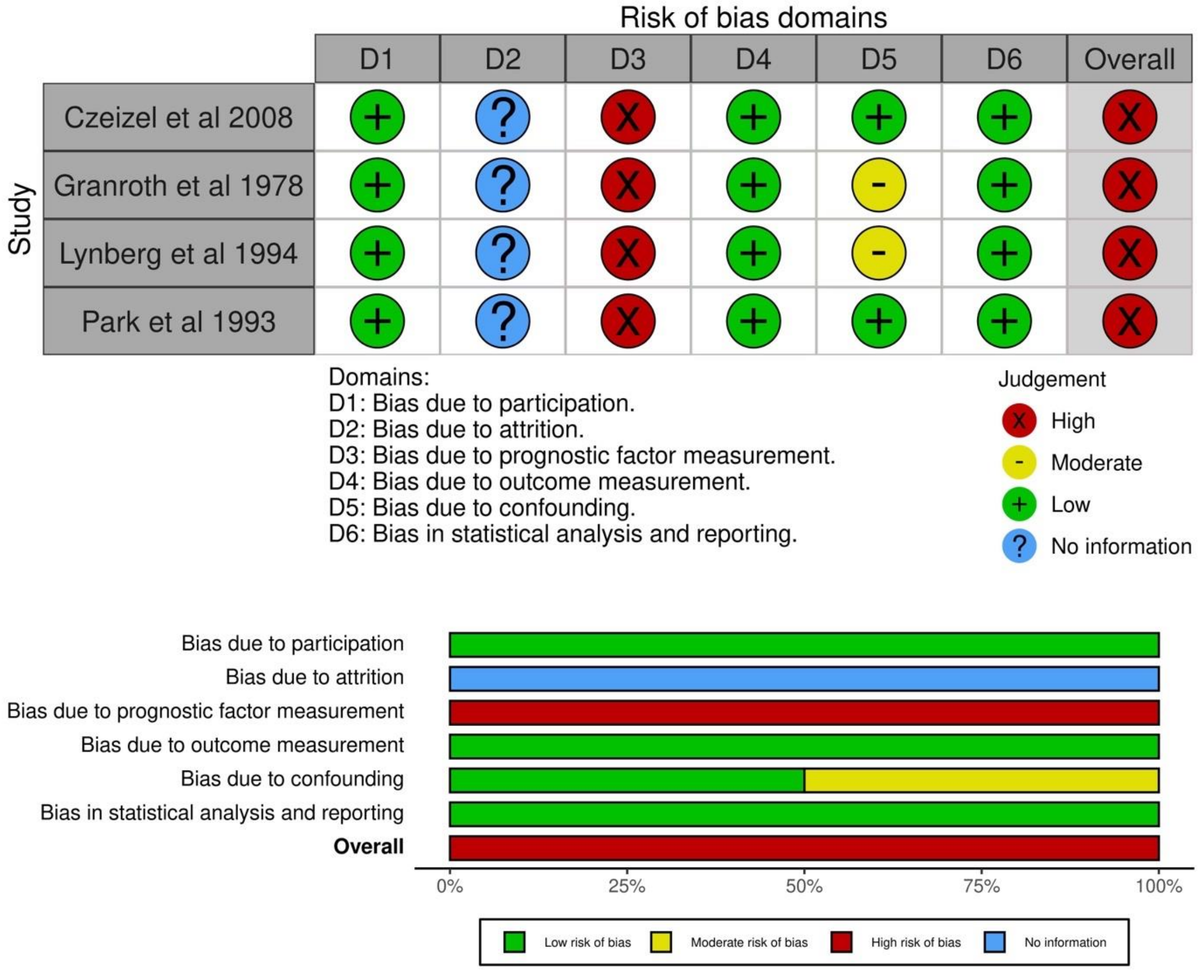

Figure S4. Risk of bias assessment at study and at domain level for all neural tube defects

Figure S5. Risk of bias assessment at study and at domain level for neural tube defects in the systematic review

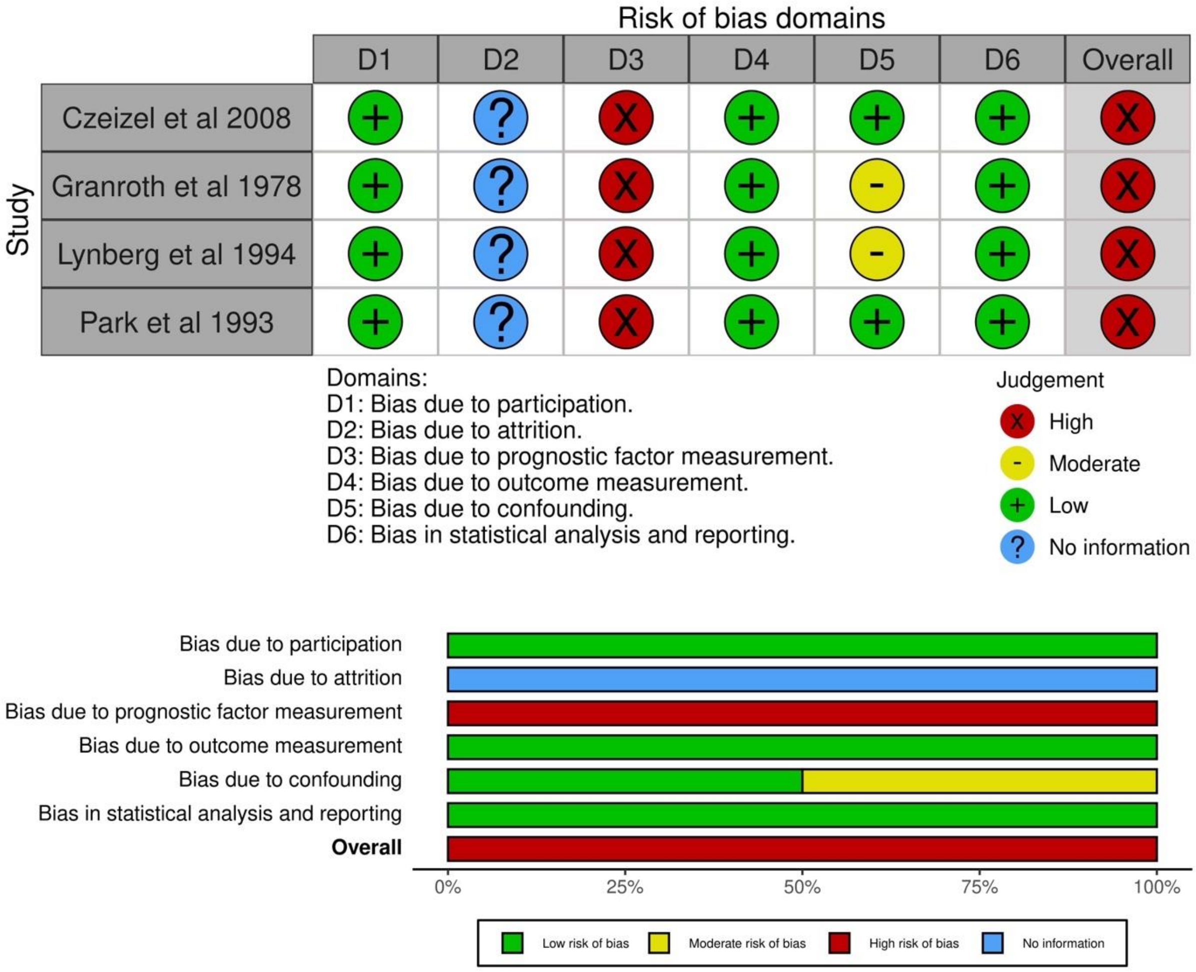

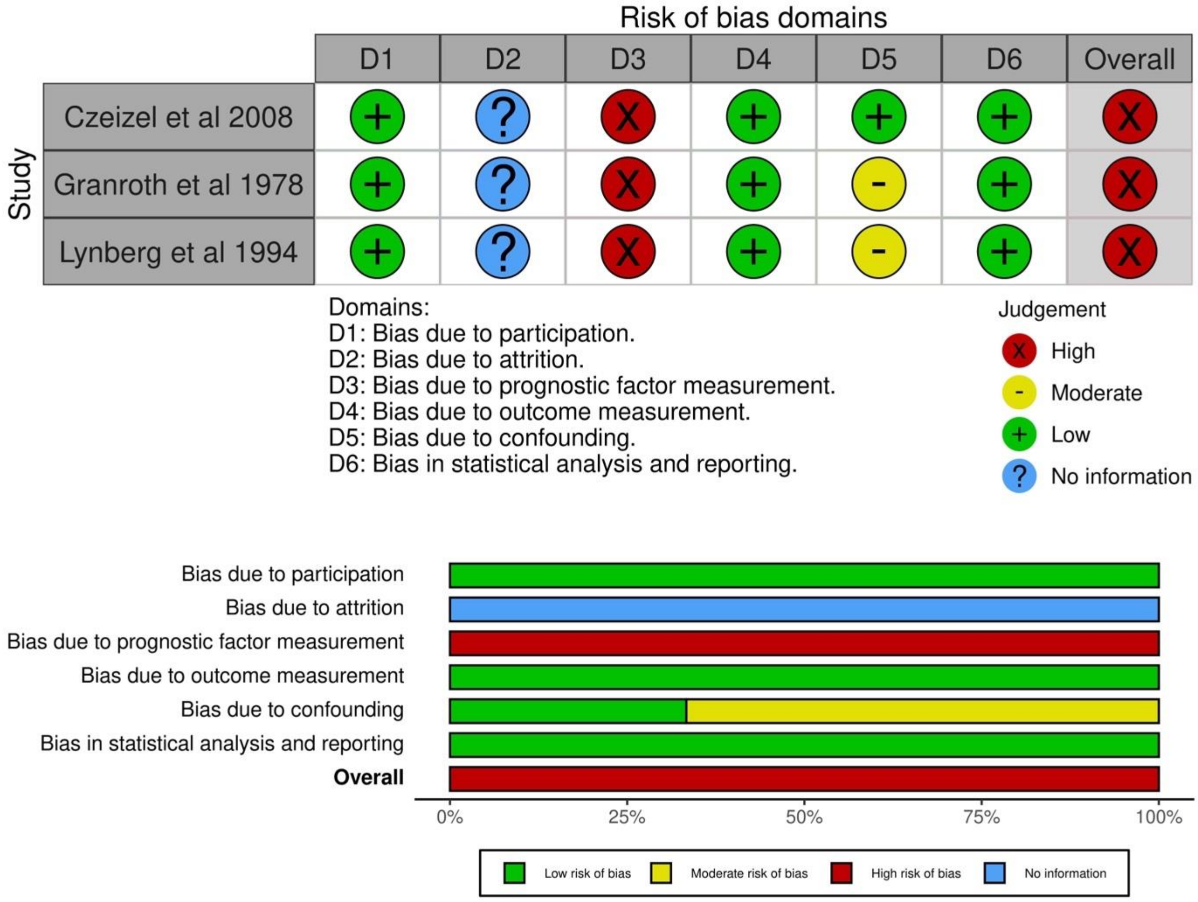

Figure S6. Risk of bias assessment at study and at domain level for spina bifida

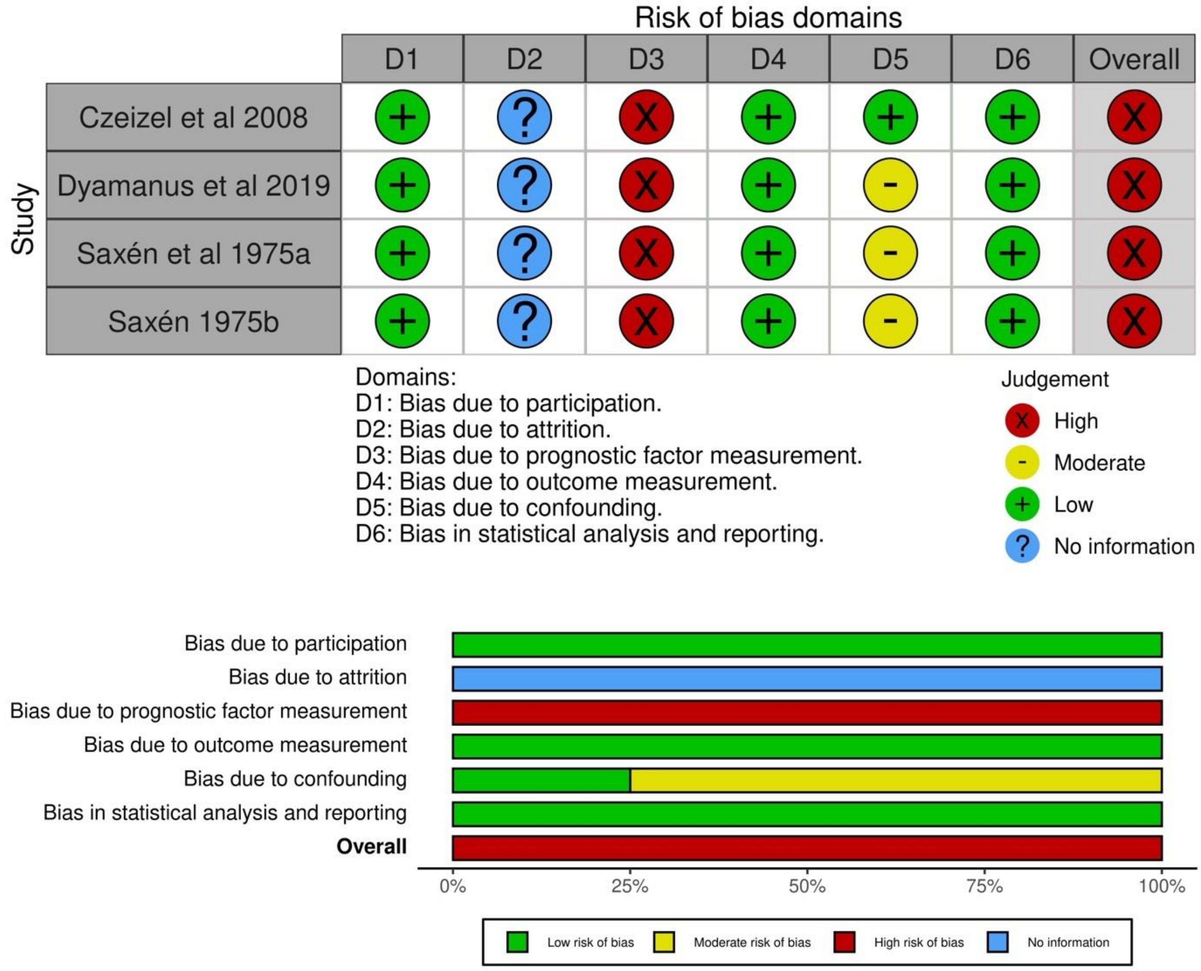

Figure S7. Risk of bias assessment at study and at domain level for cleft lip and palate

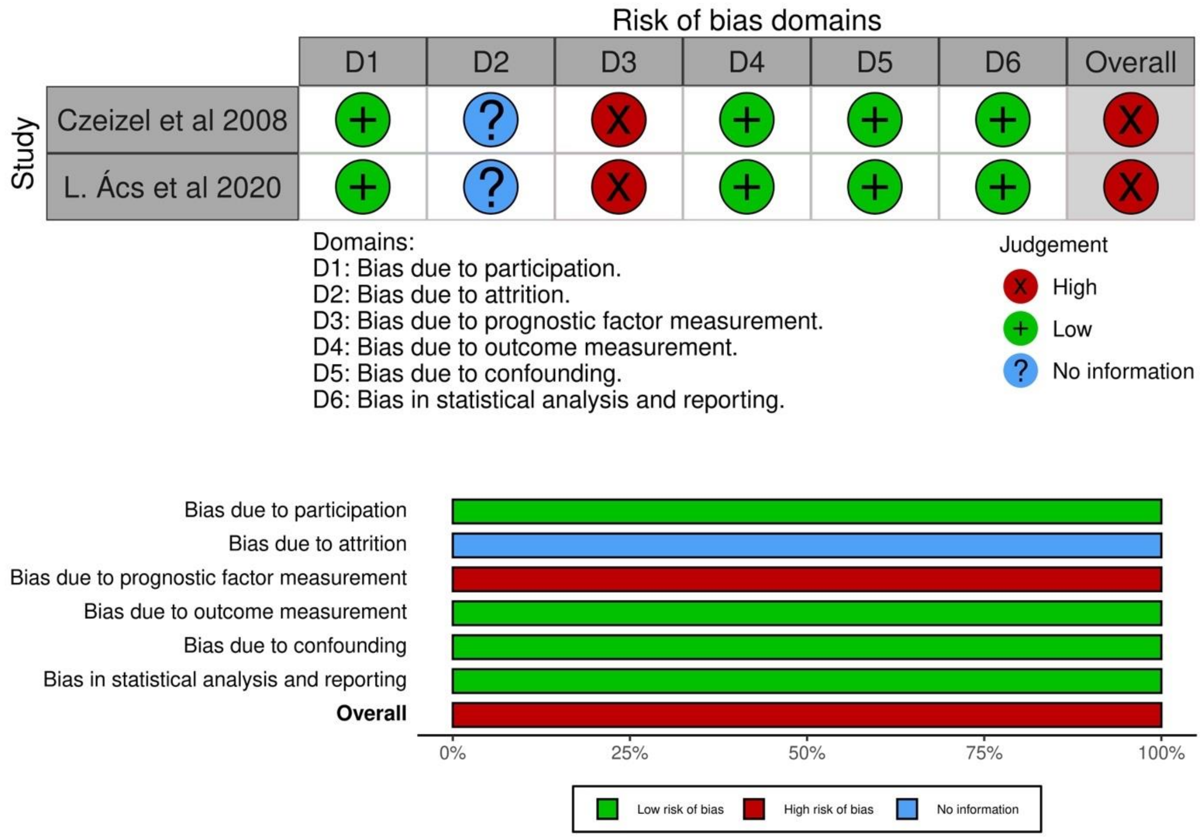

Figure S8. Risk of bias assessment at study and at domain level for cleft lip, palate

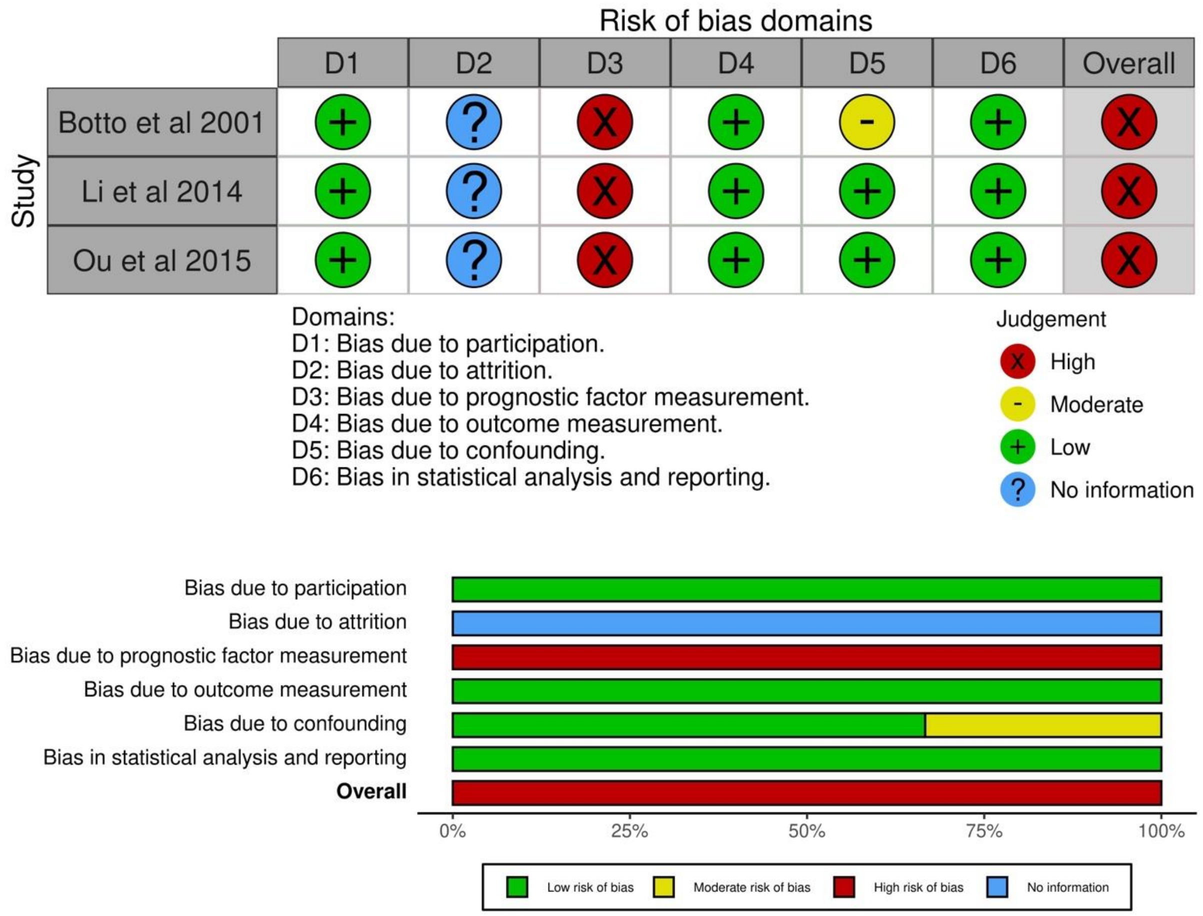

Figure S9. Risk of bias assessment at study and at domain level for congenital heart disease in the systematic review (adjusted)

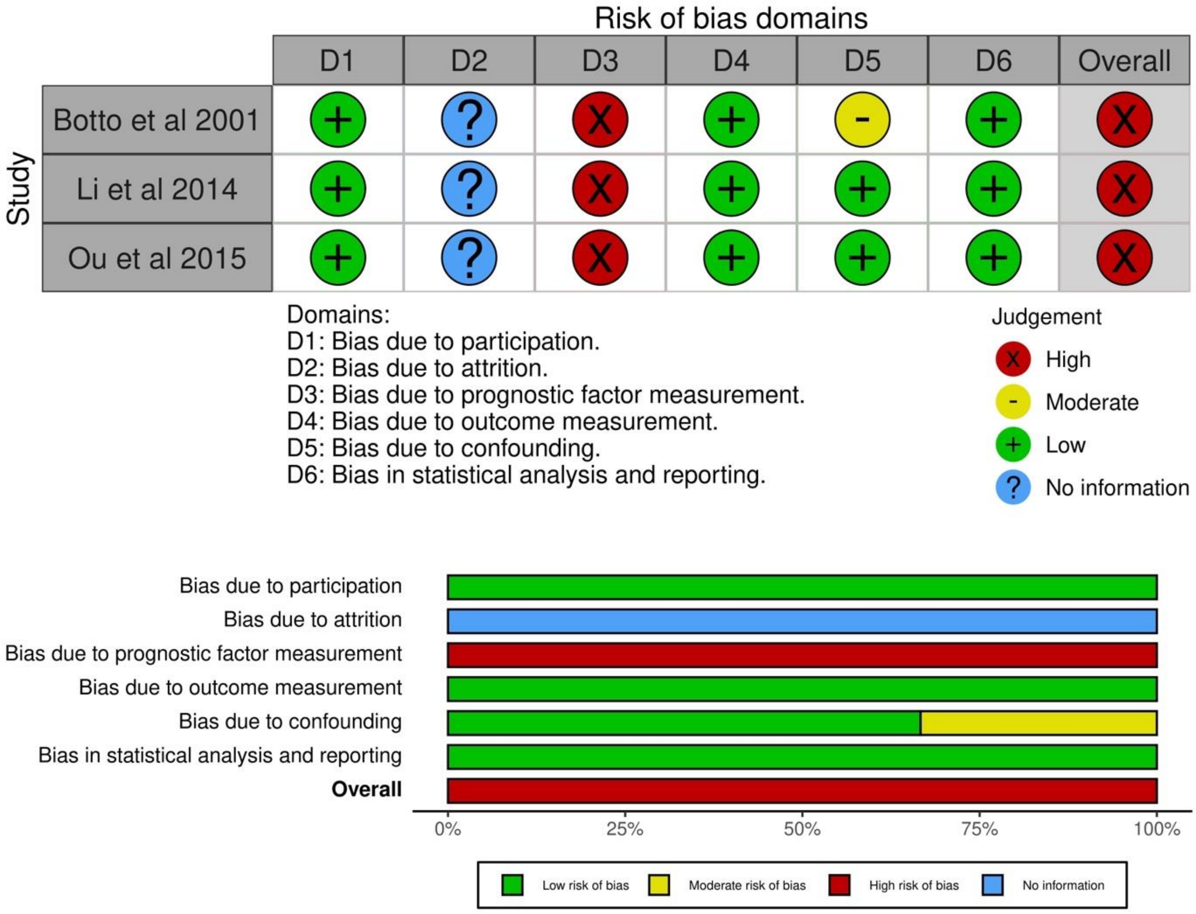

Figure S10. Risk of bias assessment at study and at domain level for congenital heart disease (adjusted)

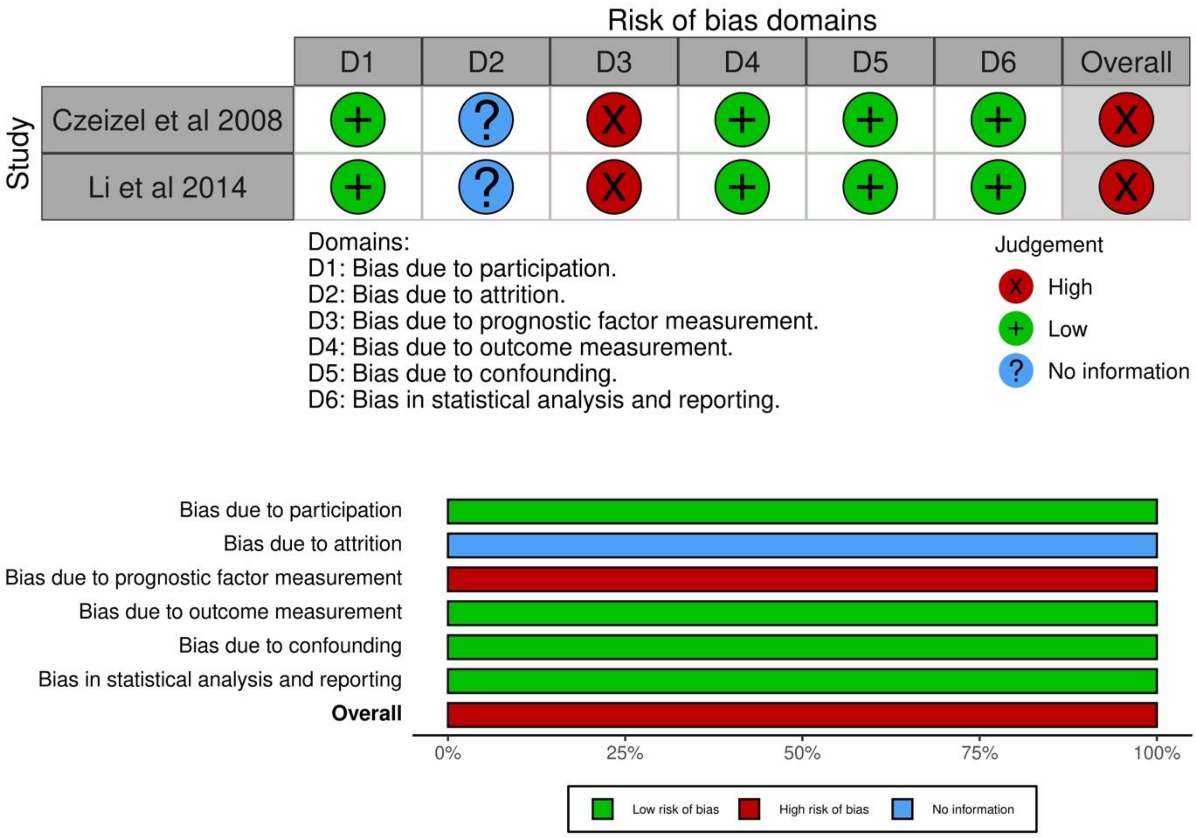

Figure S11. Risk of bias assessment at study and at domain level for congenital heart disease in the systematic review (unadjusted)

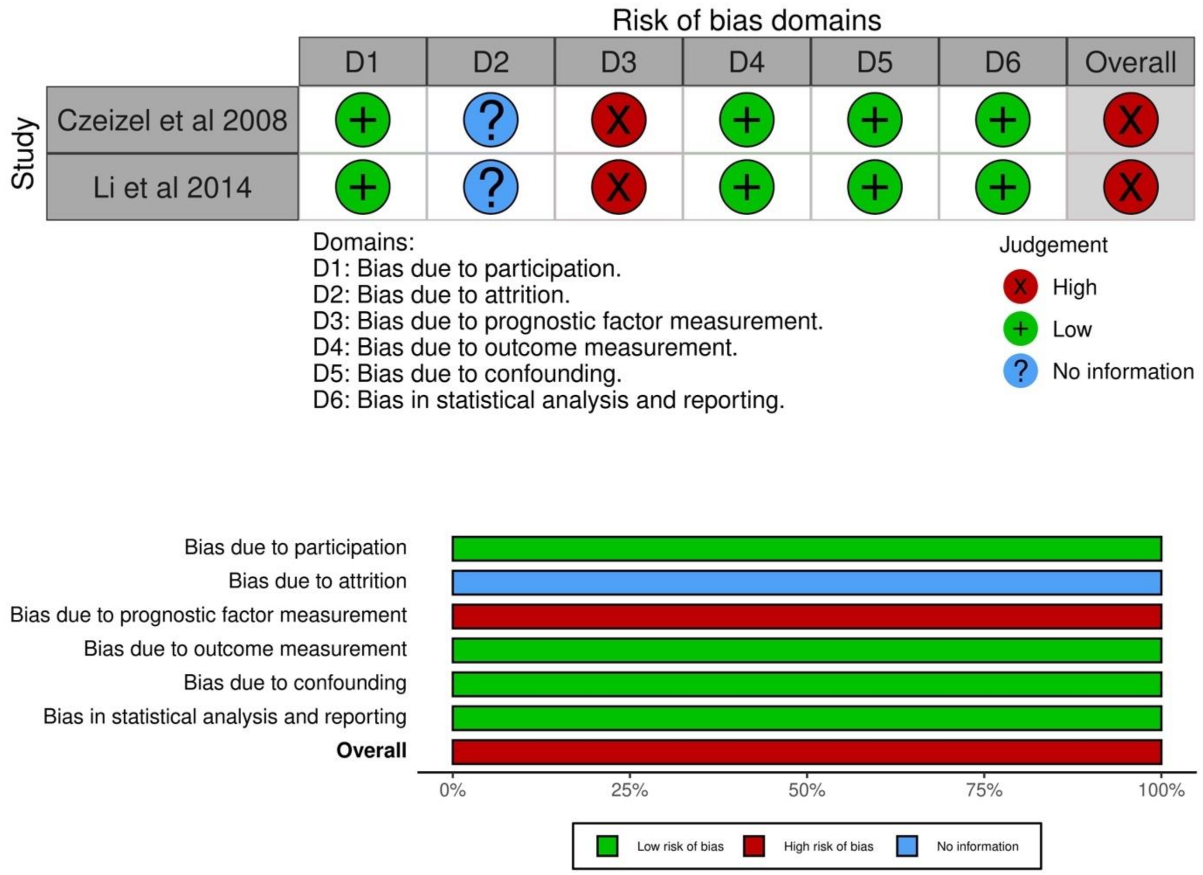

Figure S12. Risk of bias assessment at study and at domain level for congenital heart disease (unadjusted)

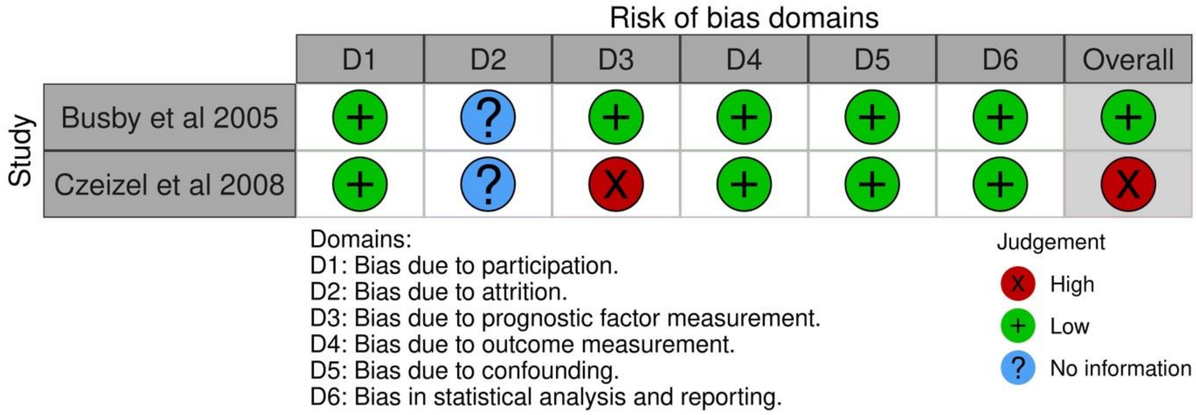

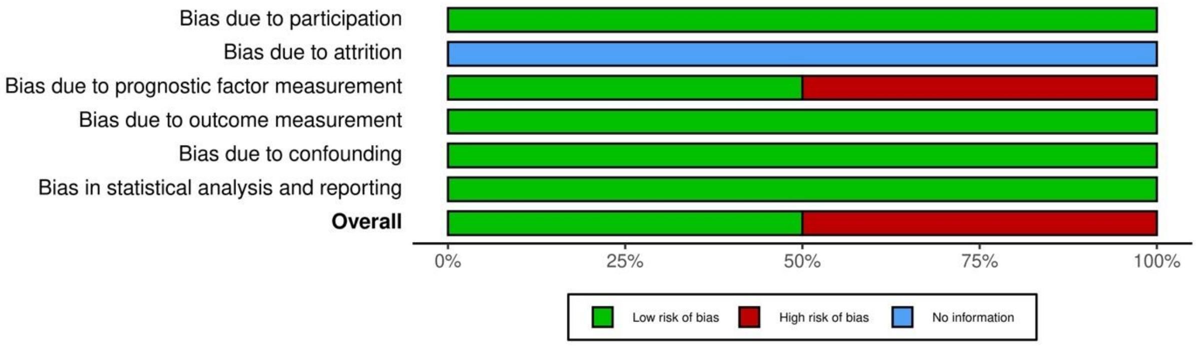

Figure S13. Risk of bias assessment at study and at domain level for eye anomalies

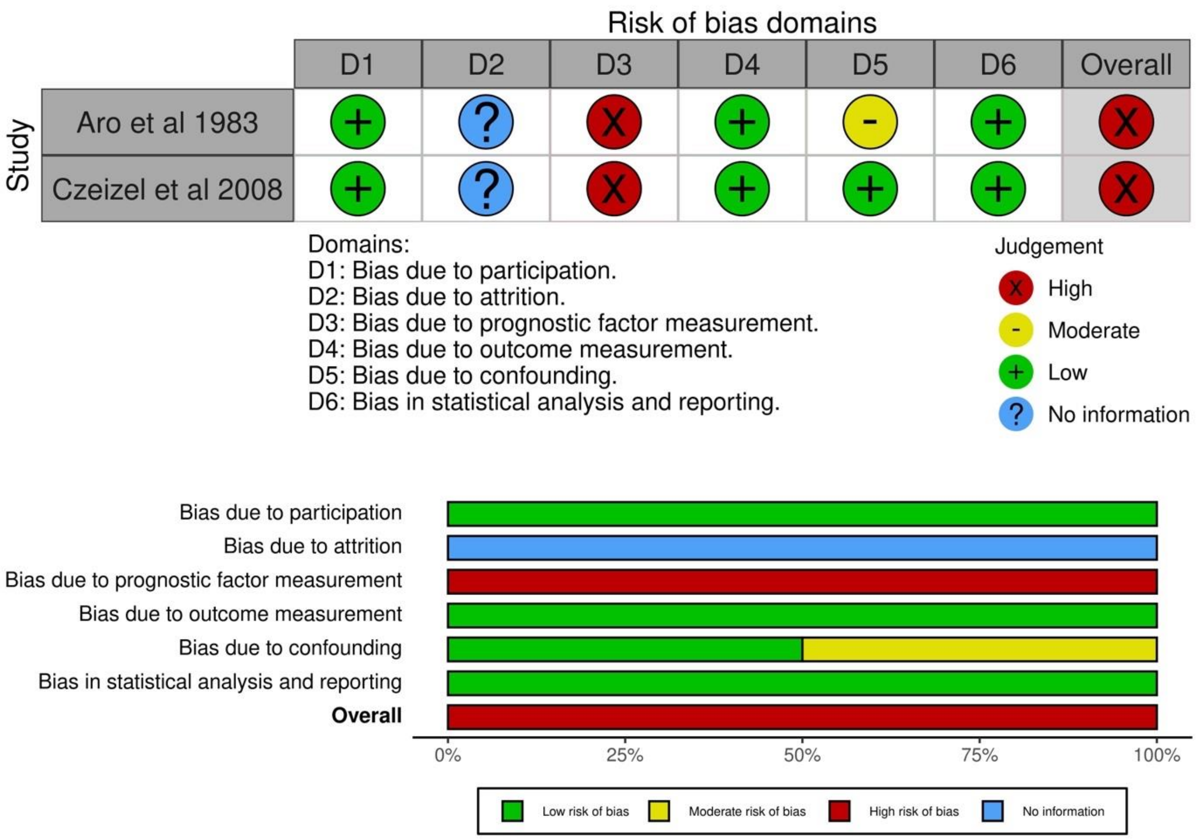

Figure S14. Risk of bias assessment at study and at domain level for limb reduction

PubMed:

(influenza OR flu) AND ((pregnant OR pregnancy) OR ((congenital OR birth) AND (anomaly OR anomalies OR abnormality OR abnormalities OR disorder OR disorders OR malformation OR malformations OR defect OR defects)))

Central:

(influenza OR flu) AND ((pregnant OR pregnancy) OR ((congenital OR birth) AND (anomaly OR anomalies OR abnormality OR abnormalities OR disorder OR disorders OR malformation OR malformations OR defect OR defects)))

Embase:

(influenza OR flu) AND ((pregnant OR pregnancy) OR ((congenital OR birth) AND (anomaly OR anomalies OR abnormality OR abnormalities OR disorder OR disorders OR malformation OR malformations OR defect OR defects)))

Figure S15. Search key of the systematic search

Table S5. Quality of evidence

Certainty assessment							Summary of findings				
							Study event rates (%)			Anticipated absolute effects	
Participants (studies) Follow-up	Risk of bias	Inconsistency	Indirectness	Imprecision	Publication bias	Overall certainty of evidence	With Risk of CA without Influenza	With Risk of CA with influenza	Relative effect (95% CI)	Risk with Risk of CA without Influenza	Risk difference with Risk of CA with influenza
**Neural tube defects**											
0 cases 0 controls (4 observational studies)	very serious	not serious	not serious	not serious	strong association	⊕◯◯◯ Very low	0 cases 0 controls		**OR 2.48** (1.95 to 3.14)	**Low**	
										0 per 1 000	**0 fewer per 1 000** (from 0 fewer to 0 fewer)
**Spina bifida**											
0 cases 0 controls (3 observational studies)	very serious	not serious	not serious	not serious	strong association	⊕◯◯◯ Very low	0 cases 0 controls		**OR 2.22** (1.58 to 3.12)	**Low**	
										0 per 1 000	**0 fewer per 1 000** (from 0 fewer to 0 fewer)
**Cleft lip and palate**											
		not serious	not serious	not serious			0 cases 0 controls			**Low**	

Certainty assessment							Summary of findings			
0 cases 0 controls (4 observational studies)	very serious				strong association	⊕◯◯◯ Very low		**OR 2.48** (1.87 to 3.28)	0 per 1 000	**0 fewer per 1 000** (from 0 fewer to 0 fewer)
**Cleft lip**										
0 cases 0 controls (1 observational study)	very serious	not serious	not serious	not serious	strong association	⊕◯◯◯ Very low	0 cases 0 controls	**OR 2.40** (1.42 to 4.06)	**Low**	
									0 per 1 000	**0 fewer per 1 000** (from 0 fewer to 0 fewer)
**Cleft palate**										
0 cases 0 controls (1 observational study)	very serious	not serious	not serious	not serious	strong association	⊕◯◯◯ Very low	0 cases 0 controls	**OR 2.95** (1.75 to 4.95)	**Low**	
									0 per 1 000	**0 fewer per 1 000** (from 0 fewer to 0 fewer)
**Congenital heart defects (adjusted)**										
0 cases 0 controls (3 observational studies)	very serious	not serious	not serious	not serious	none	⊕◯◯◯ Very low	0 cases 0 controls	**OR 1.63** (0.33 to 8.17)	**Low**	
									0 per 1 000	**0 fewer per 1 000** (from 0 fewer to 0 fewer)
**Congenital heart defects (unadjusted)**										
		not serious	not serious	not serious	none		0 cases 0 controls		**Low**	

Certainty assessment							Summary of findings			
0 cases 0 controls (2 observational studies)	very serious					⊕◯◯◯ Very low		**OR 1.80** (1.49 to 2.18)	0 per 1 000	**0 fewer per 1 000** (from 0 fewer to 0 fewer)
**Eye anomalies**										
0 cases 0 controls (2 observational studies)	serious	not serious	not serious	not serious	none	⊕◯◯◯ Very low	0 cases 0 controls	**OR 1.26** (1.02 to 1.56)	**Low**	
									0 per 1 000	**0 fewer per 1 000** (from 0 fewer to 0 fewer)
**Limb reduction defects**										
0 cases 0 controls (2 observational studies)	very serious	not serious	not serious	not serious	strong association	⊕◯◯◯ Very low	0 cases 0 controls	**OR 2.13** (1.43 to 3.18)	**Low**	
									0 per 1 000	**0 fewer per 1 000** (from 0 fewer to 0 fewer)

References

1. Ács, L., et al., *Maternal-related factors in the origin of isolated cleft palate-A population-based case-control study*. Orthod Craniofac Res, 2020. **23**(2): p. 174-180.
2. Acs, N., et al., *Maternal influenza during pregnancy and risk of congenital abnormalities in offspring*. Birth Defects Res A Clin Mol Teratol, 2005. **73**(12): p. 989-96.
3. Aro, T., *Maternal diseases, alcohol consumption and smoking during pregnancy associated with reduction limb defects*. Early Hum Dev, 1983. **9**(1): p. 49-57.
4. Botto, L.D., M.C. Lynberg, and J.D. Erickson, *Congenital heart defects, maternal febrile illness, and multivitamin use: A population-based study*. Epidemiology, 2001. **12**(5): p. 485-490.
5. Busby, A., H. Dolk, and B. Armstrong, *Eye anomalies: seasonal variation and maternal viral infections*. Epidemiology, 2005. **16**(3): p. 317-22.
6. Czeizel, A.E., et al., *Use of specified critical periods of different congenital abnormalities instead of the first trimester concept*. Birth Defects Res A Clin Mol Teratol, 2008. **82**(3): p. 139-46.
7. Dymanus, K., et al., *The association of influenza on the incidence of orofacial clefts*. Cleft Palate-Craniofacial Journal, 2019. **56**(1): p. 61-62.
8. Granroth, G., *Defects of the central nervous system in Finland: III. Disease and drugs in pregnancy*. Early Hum Dev, 1978. **2**(2): p. 147-62.
9. Li, M., et al., *Maternal influenza-like illness, medication use during pregnancy and risk of congenital heart defects in offspring*. J Matern Fetal Neonatal Med, 2014. **27**(8): p. 807-11.
10. Lynberg, M.C., et al., *Maternal flu, fever, and the risk of neural tube defects: a population-based case-control study*. Am J Epidemiol, 1994. **140**(3): p. 244-55.
11. Ou, Y., et al., *Risk factors of different congenital heart defects in Guangdong, China*. Pediatr Res, 2016. **79**(4): p. 549-58.
12. Park, C.H., et al., *Is there etiologic heterogeneity between upper and lower neural tube defects?* American Journal of Epidemiology, 1993. **136**(12): p. 1493-1501.
13. Saxén, I., *Epidemiology of cleft lip and palate. An attempt to rule out chance correlations*. Br J Prev Soc Med, 1975. **29**(2): p. 103-10.
14. Saxén, I., *The association between maternal influenza, drug consumption and oral clefts*. Acta Odontol Scand, 1975. **33**(5): p. 259-67.
